# Comparison of Anti-Inflammatory Effects of Flavonoid-Rich Common and Tartary Buckwheat Sprout Extracts in Lipopolysaccharide-Stimulated RAW 264.7 and Peritoneal Macrophages

**DOI:** 10.1155/2017/9658030

**Published:** 2017-08-27

**Authors:** Tae Gyu Nam, Tae-Gyu Lim, Bong Han Lee, Sol Lim, Hee Kang, Seok Hyun Eom, Miyoung Yoo, Hae Won Jang, Dae-Ok Kim

**Affiliations:** ^1^Korea Food Research Institute, Seongnam 13539, Republic of Korea; ^2^Department of Food Science and Biotechnology, Kyung Hee University, Yongin 17104, Republic of Korea; ^3^Graduate School of East-West Medical Science, Kyung Hee University, Yongin 17104, Republic of Korea; ^4^Department of Horticultural Biotechnology, Kyung Hee University, Yongin 17104, Republic of Korea

## Abstract

Buckwheat sprouts have been widely consumed all around world due to their great abundance of bioactive compounds. In this study, the anti-inflammatory effects of flavonoid-rich common buckwheat sprout (CBS) and tartary buckwheat sprout (TBS) extracts were evaluated in lipopolysaccharide- (LPS-) stimulated RAW 264.7 murine macrophages and primary peritoneal macrophages from male BALB/c mice. Based on the reversed-phase HPLC analysis, the major flavonoids in CBS were determined to be *C*-glycosylflavones (orientin, isoorientin, vitexin, and isovitexin), quercetin-3-*O*-robinobioside, and rutin, whereas TBS contained only high amounts of rutin. The TBS extract exhibited higher inhibitory activity as assessed by the production of proinflammatory mediators such as nitric oxide and cytokines including tumor necrosis factor-*α*, interleukin- (IL-) 6, and IL-12 in LPS-stimulated RAW 264.7 macrophages than CBS extract. In addition, TBS extract suppressed nuclear factor-kappa B activation by preventing inhibitor kappa B-alpha degradation and mitogen-activated protein kinase phosphorylation in LPS-stimulated RAW 264.7 macrophages. Moreover, the TBS extract markedly reduced LPS-induced cytokine production in peritoneal macrophages. Taken together, these findings suggest that TBS extract can be a potential source of anti-inflammatory agents that may influence macrophage-mediated inflammatory disorders.

## 1. Introduction

Inflammation encompasses cellular, immune, and metabolic responses that protect the body from injury and infection. Macrophages are extraordinarily versatile cells that play a central role in the first-line inflammatory responses by regulating protective reactions. In normal circumstances, self-limiting inflammatory responses emerge following the inhibition of inflammatory mediator [1]. However, excessive or prolonged inflammatory responses induced by macrophages are a major risk factor for chronic inflammatory diseases, including atherosclerosis, obesity, systemic lupus erythematosus, and diabetes [2].

Macrophages activated by lipopolysaccharide (LPS) can enhance the production of inflammatory mediators and cytokines, including nitric oxide (NO), inducible nitric oxide synthase (iNOS), cyclooxygenase- (COX-) 2, interleukin- (IL-) 6, IL-12, and tumor necrosis factor- (TNF-) *α* [3]. The production of proinflammatory markers is closely linked to the activation of nuclear factor-kappa B (NF-*κ*B) and mitogen-activated protein kinases (MAPKs) in macrophages [4]. NF-*κ*B, a primary transcription factor, regulates the expression of various cellular gene-encoding factors for inflammatory responses. The MAPK signaling pathways, which include extracellular signal-regulated kinase (ERK), c-Jun N-terminal kinase (JNK), and p38, are known to induce COX-2 and iNOS expression in LPS-stimulated macrophage [5]. Moreover, Guha and Mackman [6] have reported that MAPKs play a critical role in the activation of the NF-*κ*B signaling pathway. Therefore, compounds that regulate both the NF-*κ*B and MAPKs pathways have the potential to prevent inflammatory diseases.

Buckwheat (*Fagopyrum* spp.) belongs to the Polygonaceae family and two types of buckwheat called common buckwheat (*F. esculentum* Möench) and tartary buckwheat (*F. tataricum* Gaertn.) are widely used. Buckwheat sprouts have become a popular nutritional food as they have been reported to contain a greater abundance of amino acids, vitamins, and flavonoids than seeds [7]. As a result of these benefits, in recent years, buckwheat sprouts have been widely consumed as raw vegetable all around world. A previous study has reported that common buckwheat sprouts (CBS) contain an abundance of flavonoids, including *C*-glycosylflavones (orientin, vitexin, and their isomers) and rutin, whereas tartary buckwheat sprouts (TBS) contain only high concentrations of rutin [7]. Some flavonoids in buckwheat sprouts appear to have beneficial effects on human health, particularly with regard to the inhibition of inflammatory process [8, 9]. Ishii et al. [10] have reported the anti-inflammatory effects of buckwheat sprouts on human colon cancer cells. However, the anti-inflammatory effects of CBS and TBS extracts using RAW 264.7 and primary peritoneal macrophages have rarely been reported. Moreover, to the best of our knowledge, the anti-inflammatory effects of CBS and TBS extracts, which contain different compositions and amounts of flavonoids, have not been directly compared in an inflammatory context.

The objective of the present study was to comparatively investigate the effects of CBS and TBS extracts on LPS-induced inflammation in RAW 264.7 and peritoneal macrophages. The flavonoid composition of two types of buckwheat sprouts was also simultaneously analyzed using reversed-phase high-performance liquid chromatography (HPLC).

## 2. Materials and Methods

### 2.1. Materials

Primary antibodies against *β*-actin, JNK, ERK, and p38 were purchased from Santa Cruz Biotechnology (Santa Cruz, CA, USA). The iNOS, COX-2, and phosphorylated-I*κ*B-*α*, phosphorylated-JNK, phosphorylated-ERK, phosphorylated-p38, and phosphorylated-MKK4 antibodies were obtained from Cell Signaling Technology (Danvers, MA, USA). A protein assay kit was obtained from Bio-Rad Laboratories (Hercules, CA, USA). Orientin, isoorientin, vitexin, isovitexin, rutin, 3-(4,5-dimethylthiazol-2-yl)-2,5-diphenyltetrazolium bromide (MTT), LPS, interferon- (IFN-) *γ*, and Griess reagent were purchased from Sigma-Aldrich Co. LLC (St. Louis, MO, USA). Dulbecco's modified Eagle's medium (DMEM), fetal bovine serum (FBS), and penicillin/streptomycin were purchased from Welgene Inc. (Daegu, Republic of Korea). All solvents used were of HPLC grade.

### 2.2. Cultivation of Buckwheat Sprouts

Seeds of common and tartary buckwheat were provided by a seed company in Gwangju, Republic of Korea. Dry seeds were soaked in distilled water for 4 h at room temperature. Seeds were placed into a dark chamber for 48 h to accelerate germination. Germinated seeds were planted in dark growth chamber for 5 days. The CBS and TBS were harvested at 5 days after germination and dried in a 30°C dry oven.

### 2.3. Extraction of Flavonoids in Buckwheat Sprouts

Buckwheat sprouts (10 g) and 80% (*v*/*v*) aqueous methanol (150 mL) were mixed in a round-bottomed flask. The mixture was extracted using a homogenizer (Polytron PT 2100; Kinematica AG, Littau/Lucerne, Switzerland) for 10 min, followed by sonication for 20 min. The mixture was filtered through Whatman number 2 filter paper (Whatman International Limited, Kent, England). The filtrate was evaporated using a rotary evaporator at 40°C and further freeze-dried. The extraction yields of CBS and TBS were approximately 40.6 and 41.9% based on dry weight, respectively. The final extract was stored at −20°C prior to use. All experiments were performed in triplicate from three independent experiments.

### 2.4. HPLC Analysis of Flavonoids

**Figure 1:**
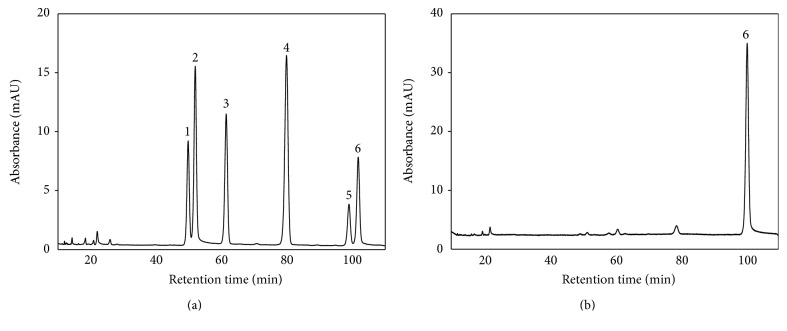
HPLC chromatograms of flavonoids in (a) common and (b) tartary buckwheat sprouts at 350 nm. Peak number 1, orientin; 2, isoorientin; 3, vitexin; 4, isovitexin; 5, quercetin-3-*O*-robinobioside; 6, rutin.

HPLC analysis of flavonoids in sprouts was carried out using a Shimadzu HPLC system (Kyoto, Japan) equipped with a photodiode array detector. HPLC conditions including linear solvent gradient of binary mobile phases, column, injection volume, and wavelength of detector were applied according to our previous study [11]. The contents of individual flavonoids were determined using authentic standard curves. HPLC chromatograms of flavonoids in buckwheat sprouts were illustrated in Figure 1.

### 2.5. RAW 264.7 Macrophages

The RAW 264.7 cell line was obtained from Korean Cell Line Bank (Seoul, Republic of Korea). The cells were cultured in DMEM supplemented with 10% FBS, 100 units/mL of penicillin, and 100 *μ*g/mL of streptomycin. Cells were maintained in a humidified atmosphere with 5% CO_2_ at 37°C.

### 2.6. Mouse Peritoneal Macrophages

Male BALB/c mice (8 weeks of age) were provided by the Korean branch of Taconic, Samtaco (Osan, Republic of Korea). All mice were maintained under pathogen-free conditions in a humidity- and temperature-controlled facility. Mice were injected intraperitoneally with 2 mL of 4% sterile thioglycollate medium (Becton Dickinson, Sparks, MD, USA). After three days, peritoneal macrophages were isolated by peritoneal gavage with 8 mL DMEM medium. The peritoneal macrophages were centrifuged and resuspended in DMEM containing 10% FBS and antibiotics. Peritoneal macrophages were then seeded in 96- and 48-well plates for evaluation of cytotoxicity and cytokine expression in cell supernatants, respectively.

### 2.7. MTT Assay for Cytotoxicity

Cytotoxicity by pretreatment with buckwheat sprouts was determined using the MTT assay. Cells were plated in 96-well plate at density of 6 × 10^4^ cells/well for peritoneal macrophages and 2 × 10^4^ cells/well for RAW 264.7 cells. The plates were incubated for 24 h prior to sample treatment. The cells were pretreated with various concentrations of samples for 24 h and cytotoxicity was determined by MTT formazan formation from viable cells. The absorbance was measured using a microplate reader (Molecular Devices, Sunnyvale, CA, USA) at 570 nm with reference wavelength at 630 nm.

### 2.8. Cellular Release of Nitric Oxide (NO)

The production of nitrite was estimated using Griess reagent (1 : 1 of 0.1% *N*-(1-naphthyl)ethylenediamine in 5% phosphoric acid and 1% sulfanilamide in 5% phosphoric acid). RAW 264.7 cells (2 × 10^5^ cells/mL) were seeded in 24-well plates and incubated for 24 h. Cells were primed with IFN-*γ* (10 ng/mL) for 1 h prior to treatment with each extract and LPS (1 *μ*g/mL) for 24 h. Equal volumes of Griess reagent and the cultured media were incubated at room temperature for 10 min. The absorbance was then measured at 540 nm using an Infinite M200 (Tecan Austria GmbH, Grödig, Austria).

### 2.9. Cytokine Determination

The anti-inflammatory effects of the buckwheat sprout extracts were evaluated based on their inhibitory effect on the production of IL-6, IL-12, and TNF-*α* in LPS-induced RAW 264.7 and peritoneal macrophages. Cells were seeded into 48-well plate at 2 × 10^5^ cells/well and incubated for 24 h. Each well was treated with various concentrations of buckwheat sprout extracts together with LPS for 24 h. The concentrations of 100 ng/mL and 1 *μ*g/mL of LPS were added for peritoneal macrophages and RAW 264.7 cells, respectively. The medium was collected and cytokines were measured using a commercially available ELISA kit according to the manufacturer's instruction (BD Pharmingen, San Diego, CA, USA).

### 2.10. cDNA Preparation and Real-Time RT-PCR

RAW 264.7 cells (5 × 10^4^ cells/well) were seeded in 24-well plates. After reaching approximately 70% confluence, each well was treated with various concentrations of buckwheat sprout extracts together with LPS (1 *μ*g/mL). After 4 h incubation, total RNA was isolated using TRIzol reagent (Invitrogen, Carlsbad, CA, USA). Real-time RT-PCR was performed with an iCycler iQ® (Bio-Rad Laboratories) using SYBR Green PCR master mix (Thermo Fisher Scientific, Waltham, MA, USA). PCR amplification was carried out using the following primers: IL-6, forward (5′-CCT CTG GTC TTC TGG AGT ACC-3′) and reverse (5′-ACT CCT TCT GTG ACT CCA GC-3′); IL-12, forward (5′-CAC CCT TGC CCT CCT AAA CC-3′) and reverse (5′-CAC CTG GCA GGT CCA GAG A-3′); TNF-*α*, forward (5′-ATG AGC ACA GAA AGC ATG A-3′) and reverse (5′-AGT AGA CAG AAG AGC GTG GT-3′); and mouse housekeeping gene glyceraldehyde 3-phosphate dehydrogenase (GAPDH), forward (5′-ATG TTC GTC ATG GGT GTG AAC-3′) and reverse (5′-GCA TGG ACT GTG GTC ATG AGT-3). All mRNA expression was normalized using GAPDH. Relative expression levels were calculated using the comparative method.

### 2.11. Western Blotting

RAW 264.7 macrophages were cultured in DMEM containing 10% (*v*/*v*) FBS at 70–80% confluency. The cells were then either primed or left unprimed with 10 ng/mL IFN-*γ* for 1 h, before each sample and LPS (1 *μ*g/mL) were added for 24 h. The cells were then lysed with 1× cell lysis buffer (Cell Signaling Technology), and protein concentration was measured using a Pierce™ BCA Protein Assay Kit (Thermo Fisher Scientific). Equal quantities of total protein were loaded onto a 10% SDS polyacrylamide gel (Bio-Rad Laboratories) for separation. The separated proteins were transferred to Immobilon P membranes (Millipore, Billerica, MA, USA). Nonspecific proteins were blocked with 5% fat-free milk for 1 h, before the primary antibody was treated at 4°C overnight. Protein bands were detected with a chemiluminescence detection kit (GE Healthcare, NJ, USA) after hybridization with an HRP-conjugated secondary antibody (Cell Signaling Technology). The COX-2, iNOS, and p-I*κ*B-*α* protein levels were expressed as a relative value to that of *β*-actin. P-JNK, p-ERK, p-p38, and p-MKK4 levels were expressed as a relative value to that of JNK, ERK, p38, and MKK4, respectively. Relative protein levels were quantified by using ImageJ (National Institutes of Health, Bethesda, MD, USA).

### 2.12. Statistical Analysis

Data are expressed as means ± standard deviation of three replicate determinations. Statistical analysis was performed using the unpaired Student's *t*-test or one-way analysis of variance (ANOVA) followed by Dunnett's post hoc test for multiple comparison. The *p* value of less than 0.05 was considered as a significant difference. Statistical analysis was conducted using IBM SPSS statistics software (version 20, SPSS Inc., Chicago, IL, USA).

## 3. Results

### 3.1. Quantification of Flavonoids in Buckwheat Sprouts

Flavonoids in CBS and TBS were quantitatively analyzed using a reversed-phase HPLC (Table 1). *C*-Glycosylflavones (orientin, isoorientin, vitexin, and isovitexin), quercetin-3-*O*-robinobioside and rutin were detected in CBS, whereas only rutin was detected in TBS. The quantities of individual flavonoids present in 1 g extracts of CBS were 6.4 mg orientin, 12.9 mg isoorientin, 12.1 mg vitexin, 16.4 mg isovitexin, 5.5 mg quercetin-3-*O*-robinobioside, and 11.8 mg rutin. The content of rutin (42.1 mg/g extracts) in TBS was approximately 3.6-fold higher than that in CBS.

### 3.2. Effect of Buckwheat Sprouts on LPS-Induced Expression of NO, iNOS, and COX-2 in RAW 264.7 Cells

We first investigated the cytotoxicity of the buckwheat sprout extracts to determine the applicable treatment concentration range. A decrease in cell viability by more than 10% when compared to control cells was considered to be cytotoxic. Neither the CBS nor TBS extracts were cytotoxic at the highest concentrations tested, as determined by MTT assay (Supp. Figure 1 in Supplementary Material available online at https://doi.org/10.1155/2017/9658030).

**Figure 2:**
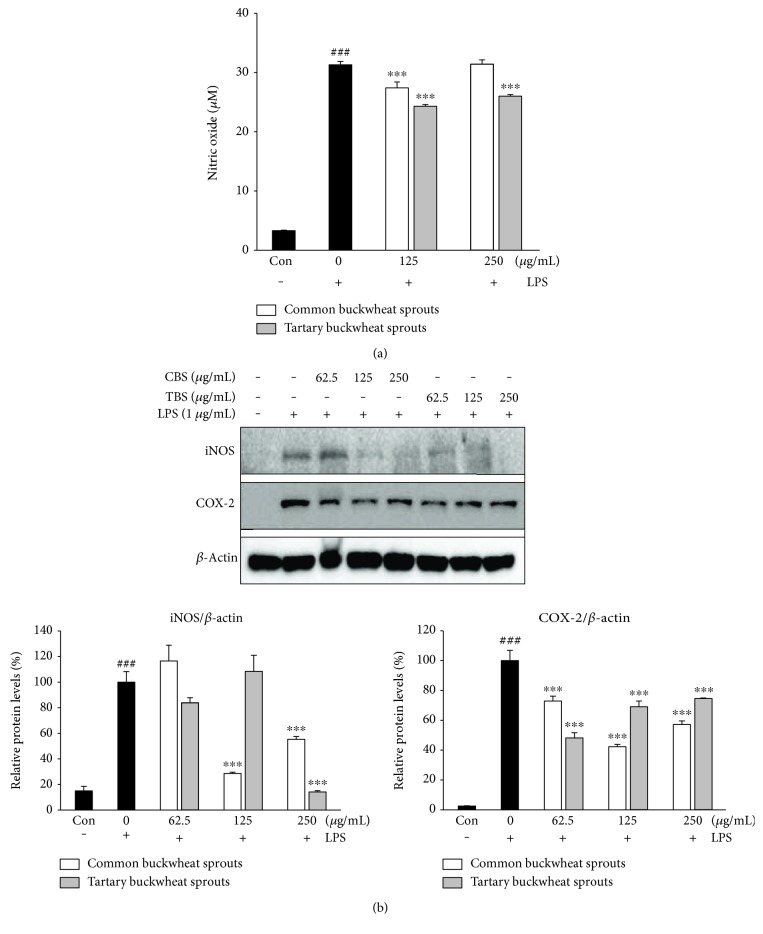
Inhibitory effects of common buckwheat sprout (CBS) and tartary buckwheat sprout (TBS) extracts on (a) NO production and (b) levels of iNOS and COX-2 in LPS-induced RAW 264.7 macrophages. RAW 264.7 macrophages were incubated for 24 h, and exposed to sprout extracts together with LPS (1 *μ*g/mL) for 24 h. Levels of iNOS and COX-2 expression were determined using Western blot assay. Relative protein levels are expressed as the percentage of intensity to the cells treated with LPS alone, which was set to 100%. Data represent the means ± standard deviation of three independent experiments. ### indicates *p* < 0.001 in comparison to untreated controls; significant difference was determined using unpaired Student's *t*-test. ∗∗∗ indicates *p* < 0.001 in comparison to cells treated with LPS alone by one-way ANOVA followed by Dunnett's test for multiple comparison.

The inhibitory effect of the sprout extracts on LPS-induced NO release in RAW 264.7 cells was assessed based on the Griess assay (Figure 2(a)). The level of NO was markedly increased after treatment with LPS for 24 h; however, TBS and CBS extracts significantly (*p* < 0.001) suppressed the release of NO compared with the control exposed to LPS only. Of particular note, treatment with CBS and TBS extracts at the same dose (125 *μ*g/mL) decreased NO production by approximately 12.5 and 22.4%, respectively, indicating that TBS extract possesses higher inhibition of NO production in RAW 264.7 cells than CBS extract. However, 250 *μ*g/mL CBS extract showed no effect on NO generation compared to control group treated with LPS only.

To determine the inhibitory effects of the sprout extracts on iNOS and COX-2 expression, Western blot analysis was also conducted with LPS-induced RAW 264.7 cells. As shown in Figure 2(b), both CBS and TBS extracts at concentration of 250 *μ*g/mL significantly (*p* < 0.001) reduced the expression of iNOS compared to control group treated with LPS only. Especially, 250 *μ*g/mL TBS extract inhibited the expression of iNOS by more than 85%. The TBS extract at concentration of 250 *μ*g/mL reduced the expression of iNOS better than CBS extract at the same dose. Moreover, both CBS and TBS extracts significantly (*p* < 0.001) reduced protein expressions of COX-2 at all concentrations tested.

### 3.3. Effect of Buckwheat Sprouts on Cytokine Production and mRNA Expression in LPS-Activated RAW 264.7 Cells

**Figure 3:**
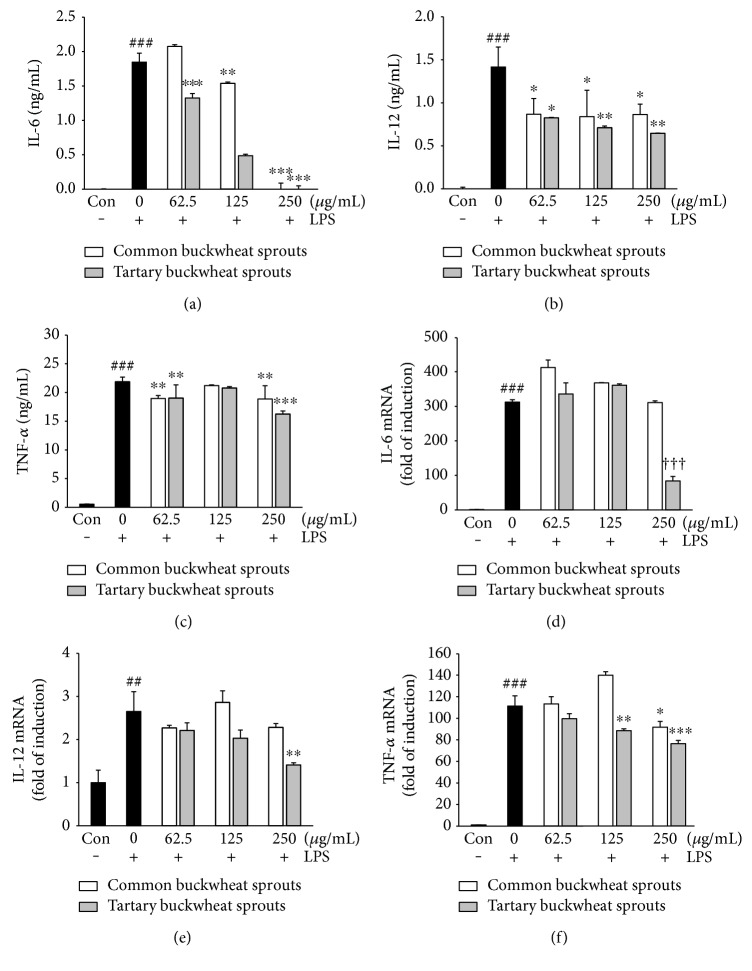
Inhibitory effect of common and tartary buckwheat sprout extracts on LPS-induced (a) IL-6, (b) IL-12, and (c) TNF-*α* cytokine production and LPS-induced (d) IL-6, (e) IL-12, and (f) TNF-*α* mRNA expression in RAW 264.7 macrophages. RAW 264.7 macrophages were exposed to sprout extracts together with LPS (1 *μ*g/mL). Level of cytokine expression in the culture media was measured using ELISA. mRNA levels were analyzed by real-time RT-PCR. Data represent the means ± standard deviation of three independent experiments. ^##^*p* < 0.01 and ^###^*p* < 0.001 in comparison with untreated controls; significant difference was determined using unpaired Student's *t*-test. ††† indicates *p* < 0.001 in comparison to cells treated with LPS alone (unpaired Student's *t*-test). ^∗^*p* < 0.05, ^∗∗^*p* < 0.01, and ^∗∗∗^*p* < 0.001 in comparison with cells treated with LPS alone by one-way ANOVA followed by Dunnett's test for multiple comparison.

The effect of CBS and TBS extracts on the expression of cytokines, including IL-6, IL-12, and TNF-*α*, was investigated using LPS-induced RAW 264.7 cells. As shown in Figures 3(a), 3(b), and 3(c), almost undetectable levels of cytokines were observed in LPS-untreated RAW 264.7 cells, whereas macrophages treated with LPS alone for 24 h significantly (*p* < 0.001) showed an enhanced release of IL-6, IL-12, and TNF-*α*. TBS extract significantly (*p* < 0.001) inhibited the secretion of IL-6 in a dose-dependent manner (Figure 3(a)). TBS extract at 62.5–125 *μ*g/mL exhibited a significantly (*p* < 0.001) higher inhibitory effect of IL-6 compared with CBS extract. Moreover, TBS extract at 125–250 *μ*g/mL was found to be a more potent inhibitor of IL-12 than CBS extract at the same dose (Figure 3(b)). The release of TNF-*α* was significantly decreased by treatment with CBS and TBS extracts. At the concentration of 250 *μ*g/mL, TBS extract significantly (*p* < 0.001) reduced TNF-*α* production compared with 250 *μ*g/mL CBS extract (Figure 3(c)). Consistent with the results, TBS extract showed an inhibitory effect on IL-6, IL-12, and TNF-*α* mRNA expression at 250 *μ*g/mL (Figures 3(d), 3(e), and 3(f)). However, CBS extract had no inhibitory effect on IL-6 and IL-12 mRNA expression at any concentration.

### 3.4. Effect of Buckwheat Sprouts on LPS-Stimulated NF-*κ*B Activation in RAW 264.7 Cells

**Figure 4:**
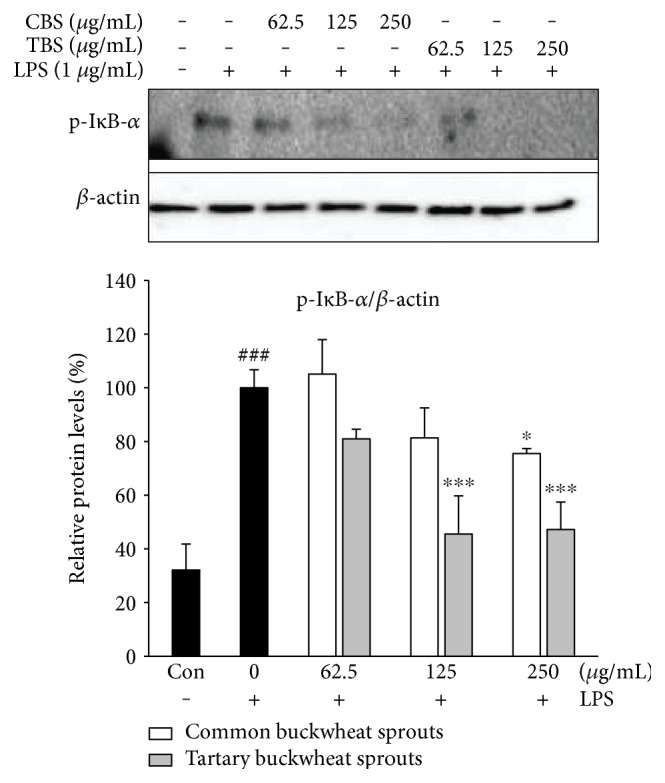
Effect of common buckwheat sprout (CBS) and tartary buckwheat sprout (TBS) extracts on LPS-stimulated phosphorylation of I*κ*B-*α* in RAW 264.7 macrophages. RAW 264.7 macrophages were incubated for 24 h and exposed to LPS (1 *μ*g/mL) with sprout extracts for 24 h. Levels of phosphorylated I*κ*B-*α* were determined using Western blot assay. Relative protein levels are expressed as the percentage of intensity to the cells treated with LPS alone, which was set to 100%. Data represent the means ± standard deviation of three independent experiments. ### indicates *p* < 0.001 in comparison with untreated controls; significant difference was determined using unpaired Student's *t*-test. ^∗^*p* < 0.05 and ^∗∗∗^*p* < 0.001 in comparison with cells treated with LPS alone by one-way ANOVA followed by Dunnett's test for multiple comparison.

The effect of CBS and TBS extracts on the activation of NF-*κ*B was investigated by analyzing the inhibition of inhibitor kappa B-alpha (I*κ*B-*α*) phosphorylation in LPS-induced RAW 264.7 cells. As shown in Figure 4, the activation of I*κ*B-*α* via phosphorylation significantly (*p* < 0.001) increased in LPS-stimulated RAW 264.7 cells, whereas CBS and TBS extracts inhibited the activation of I*κ*B-*α*. It was found that treatment with TBS extract at 125–250 *μ*g/mL significantly (*p* < 0.001) reduced the phosphorylation of I*κ*B-*α* compared to treatment with CBS extract at the same dose.

### 3.5. Effect of Buckwheat Sprouts on MAPK and MAPK Kinase (MAPKK) Phosphorylation in LPS-Stimulated RAW 264.7 Cells

**Figure 5:**
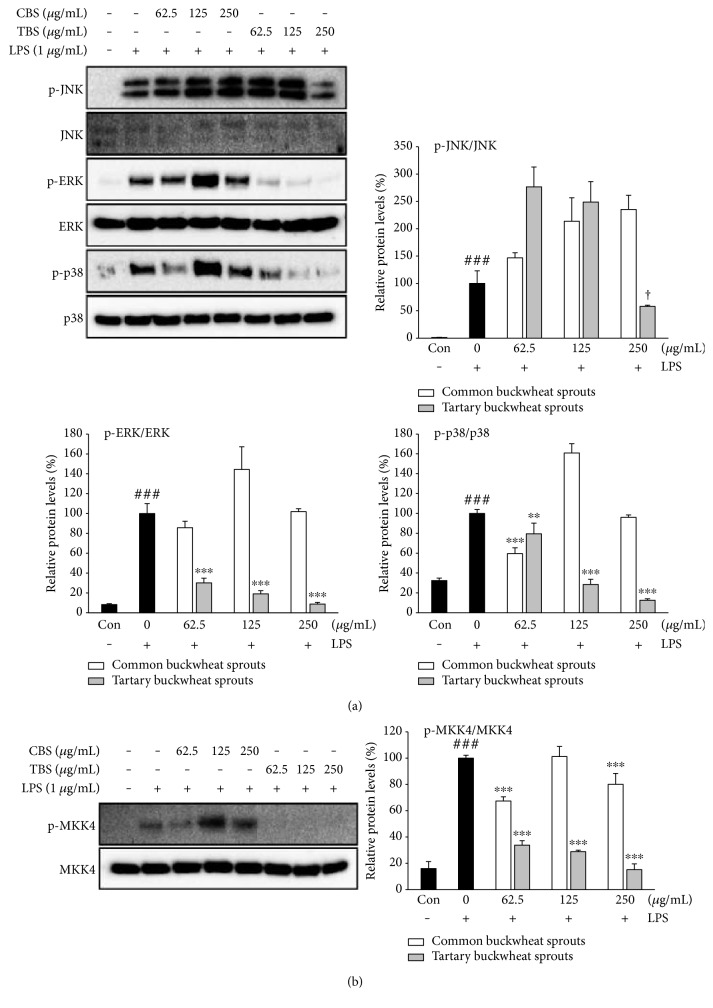
Effect of common buckwheat sprout (CBS) and tartary buckwheat sprout (TBS) extracts on LPS-stimulated (a) MAPK and (b) MAPK kinase 4 activation. RAW 264.7 macrophages were incubated for 24 h and exposed to CBS or TBS extracts with LPS (1 *μ*g/mL) for 24 h. Whole protein was determined using Western blot assay. Relative protein levels are expressed as the percentage of intensity to the cells treated with LPS alone, which was set to 100%. Data represent the means ± standard deviation of three independent experiments. † indicates *p* < 0.05 in comparison with cells treated with LPS alone (unpaired Student's *t*-test). ### indicates *p* < 0.001 in comparison with untreated controls (unpaired Student's *t*-test). ^∗∗^*p* < 0.01 and ^∗∗∗^*p* < 0.001 in comparison with cells treated with LPS alone by one-way ANOVA followed by Dunnett's test for multiple comparison.

The inhibitory effects of buckwheat sprout extracts on LPS-induced MAPKs (ERK, JNK, and p38) phosphorylation were investigated. As shown in Figure 5(a), the phosphorylation levels of JNK, ERK, and p38 were increased in LPS-induced RAW 264.7 macrophages. TBS extract at 250 *μ*g/mL significantly (*p* < 0.05) suppressed the expression of phosphorylated-JNK compared with control group treated with LPS only. Moreover, TBS extract significantly (*p* < 0.001) inhibited the expression of phosphorylated-ERK and p38 in a dose-dependent manner. CBS extract significantly (*p* < 0.001) reduced the phosphorylation level of p38 at a low concentration (62.5 *μ*g/mL), whereas the expression of phosphorylated-ERK and JNK was not inhibited by treatment with CBS extract at any concentration. Similarly, the production of phosphorylated MAPK kinase 4 (MKK4) was also obviously attenuated by TBS extract (Figure 5(b)).

### 3.6. Effect of Buckwheat Sprouts on Cytokine Production in LPS-Activated Mouse Peritoneal Macrophages

**Figure 6:**
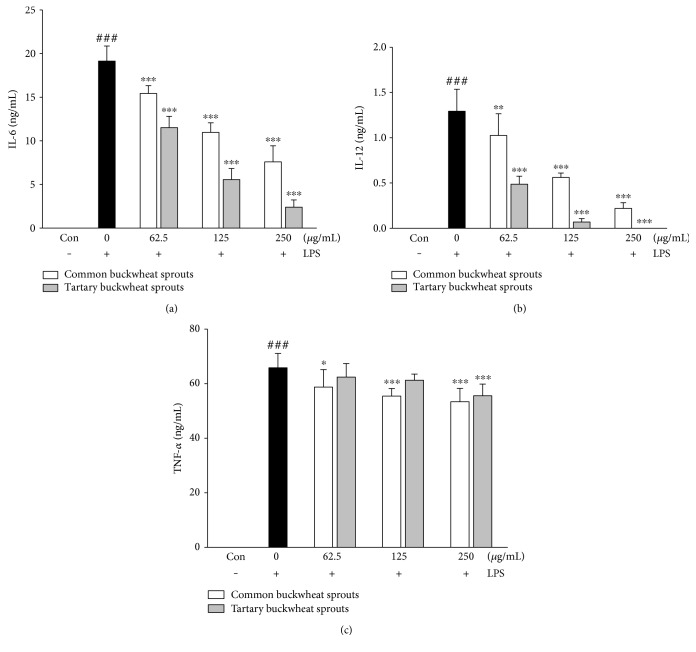
Inhibitory effects of common and tartary buckwheat sprout extracts on the secretion of (a) IL-6, (b) IL-12, and (c) TNF-*α* cytokines in LPS-induced peritoneal macrophages of male BALB/c mice. Mouse peritoneal macrophages were exposed to LPS (100 ng/mL) with sprout extracts. Levels of cytokine expression in culture media were measured by ELISA. Data represent the means ± standard deviation of three independent experiments. ### indicates *p* < 0.001 in comparison with untreated controls; significant difference was determined using unpaired Student's *t*-test. ^∗^*p* < 0.05, ^∗∗^*p* < 0.01, and ^∗∗∗^*p* < 0.001 in comparison with cells treated with LPS alone by one-way ANOVA followed by Dunnett's test for multiple comparison.

Given the results obtained concerning the anti-inflammatory activity of buckwheat sprout extracts on LPS-stimulated RAW 264.7 macrophages, we further investigated the effects of the extracts on cytokine release in peritoneal macrophages from male BALB/c mice. As shown in Figures 6(a) and 6(b), LPS significantly enhanced IL-6 and IL-12 production, while CBS and TBS extract inhibited cytokine production in a dose-dependent manner. TBS extract significantly (*p* < 0.001) reduced the secretion of IL-6 and IL-12, which was superior to CBS extract at the same dose. The production of TNF-*α* was significantly (*p* < 0.001) upregulated by LPS, and pretreatment with CBS (125–250 *μ*g/mL) and TBS (250 *μ*g/mL) significantly (*p* < 0.001) inhibited this upregulation in peritoneal macrophages (Figure 6(c)).

## 4. Discussion

Inflammation involves many complex interactions between cellular and inflammatory mediators. During the inflammatory process, significant amounts of NO and prostaglandin E2 are produced via the expression of iNOS and COX-2 [12]. The excessive production of proinflammatory mediators by macrophages can induce variety of inflammation-related disorders [3, 5]; thus, agents that suppress the production of these factors may have the potential to protect against inflammatory diseases. We investigated the inhibitory effects of two different types of buckwheat sprouts on LPS-induced proinflammatory mediators in RAW 264.7 cells (Figure 2). The results revealed that the inhibition of LPS-induced NO production by CBS and TBS extracts was accompanied by a reduction in iNOS expression. Moreover, both extracts suppressed COX-2 protein expression in LPS-stimulated RAW 264.7 macrophage. However, TBS extract (250 *μ*g/mL) was found to be more potent in reducing NO production as well as the expression of iNOS than CBS extract. Macrophage activation induced by LPS resulted in the secretion of typical proinflammatory cytokines including IL-6, IL-12, and TNF-*α* [13]. TNF-*α* is a key mediator in the inflammatory process and stimulates other cytokines such as IL-6 and IL-12. Additionally, the cytokines IL-6 and IL-12 are known to upregulate the production of iNOS in macrophages [14], while IL-12 also promotes NF-*κ*B activation in mouse peritoneal macrophages [15]. These cytokines induce the expression of iNOS and NO, and overexpression of proinflammatory mediators can result in tissue injury and multiple organ failure [16]. The upregulated IL-6, IL-12, and TNF-*α* levels in LPS-induced RAW 264.7 cells were significantly (*p* < 0.05) decreased with treatments of both CBS and TBS extracts compared with control group treated with LPS only (Figures 3(a), 3(b), and 3(c)). The TBS extract was a more potent inhibitor of the cytokines at concentrations tested in this study. Furthermore, TBS extract at concentration of 250 *μ*g/mL significantly reduced the mRNA expression of IL-6, IL-12, and TNF-*α* compared with control group treated with LPS only (Figures 3(d), 3(e), and 3(f)). Although treatment of CBS extract had no inhibitory effects on IL-6 and IL-12 mRNA expression, anti-inflammatory effects were shown by reducing the translation of IL-6 and IL-12 mRNA (Figure 3). The CBS extract may inhibit posttranscriptional pathway of IL-6 and IL-12 proteins.

LPS activates typical inflammatory signaling pathways, including the NF-*κ*B signaling pathway. NF-*κ*B is a mammalian transcription factor that regulates proinflammatory mediators and cytokines in LPS-induced macrophages and exists as a heterodimer comprising p50 and p65 subunits known to mediate the expression of genes associated with immune modulation [17]. In unstimulated macrophages, NF-*κ*B remains inactive in the cytoplasm bound to I*κ*B-*α*. The activation of NF-*κ*B by LPS stimulation occurs through phosphorylation and subsequent degradation of I*κ*B-*α*, followed by nuclear translocation of free NF-*κ*B [18]. Following activation, NF-*κ*B regulates iNOS, COX-2, and gene transcription of cytokines [19]. Our results show that TBS extract significantly (*p* < 0.001) reduced LPS-induced phosphorylation of I*κ*B-*α* (Figure 4). These results demonstrate that NF-*κ*B activation by phosphorylation of I*κ*B-*α* is suppressed by TBS extract. TBS extract had a more potent inhibitory effect on phosphorylation of I*κ*B-*α* than CBS extract (Figure 4).

The activation of MAPK pathways in macrophages is strongly associated with the inflammatory response and activates downstream proinflammatory cytokines and mediators. The MAPKs include ERK, p38, and JNK, and their signaling pathways play a crucial role in biological processes in addition to typical inflammatory signaling [20]. MAPK signaling can be activated by LPS, which upregulates the production of NO in RAW 264.7 cells [21]. The activation of ERK in response to LPS in turn leads to the upregulation of iNOS and proinflammatory cytokines [22]. Furthermore, LPS-induced COX-2, iNOS, and TNF-*α* expression in macrophages is regulated by p38 and JNK [19]. In this study, the phosphorylation levels of p38 and ERK were significantly (*p* < 0.001) attenuated by treatment of TBS extract compared with RAW 264.7 cells treated with LPS only (Figure 5(a)). Increased phosphorylation level of JNK was significantly (*p* < 0.05) reduced by TBS extract (250 *μ*g/mL) treatment. However, the CBS extract exhibited relatively weak or no effect on inhibition of MAPK activation (Figure 5(a)). These results demonstrate that the TBS extract is a more potent inhibitor of MAPK signaling, resulting in a greater reduction of proinflammatory mediators and cytokines than the CBS extract. Furthermore, the phosphorylation of MAPK family members is known to require the activated MAPKK which can phosphorylate threonine and tyrosine residues. MKK4, a member of the MAPKK, regulates the activation of p38 and JNK MAPK in response to proinflammatory cytokines [23]. To investigate the upstream kinase of p38 and JNK, the inhibitory effects of the sprout extracts on LPS-induced phosphorylation of MKK4 were examined. We were able to detect TBS extract-mediated suppression of LPS-induced MKK4 activation (Figure 5(b)). As shown in Figure 5(a), the reduction of phosphorylated-JNK and phosphorylated-p38 in response to TBS extract could be mediated via the inhibition of MKK4 activation. These results suggest that the anti-inflammatory effects of TBS extract in LPS-induced RAW 264.7 cells are likely related to suppression of the p38/JNK/ERK MAPKs and MKK4 signaling pathways.

We further investigated the anti-inflammatory effects of the extracts of buckwheat sprouts on LPS-induced peritoneal macrophages of male BALB/c mice. Similar to the results observed in RAW 264.7 cell lines, our data revealed that the CBS and TBS extracts caused a dose-dependent reduction in IL-6 and IL-12 production in LPS-induced peritoneal macrophages (Figures 6(a) and 6(b)). The production of TNF-*α* was significantly (*p* < 0.001) reduced by treatment with both CBS and TBS extracts at concentration of 250 *μ*g/mL (Figure 6(c)). The TBS extract was more potent in decreasing LPS-induced IL-6 and IL-12 production in peritoneal macrophages. The results obtained suggest that TBS extract effectively inhibits the production of cytokines in both RAW 264.7 and peritoneal macrophages.

Some flavonoids from natural products are known to inhibit and/or reduce the progression of inflammation [9]. Flavonoids appear to exhibit anti-inflammatory properties via the modulation of reactive oxygen species that induce the activation of NF-*κ*B and subsequent release of cytokines [24]. Liu et al. [25] have reported that TBS ethanol extract has higher DPPH radical scavenging, reducing power, superoxide anion scavenging activity, and directly scavenging intracellular radicals than CBS ethanol extract. We therefore speculate that TBS extract, which contains potent antioxidants, exhibits anti-inflammatory effects. In HPLC analysis, the CBS extract was found to contain *C*-glycosylflavones (orientin, vitexin, and their isomers), quercetin-3-*O*-robinobioside, and rutin, whereas the TBS extract contained rutin only (Table 1 and Figure 1). According to our findings, the CBS extract exhibited relatively weak or no effect on inhibition of inflammatory response at up to 250 *μ*g/mL which contained approximately 7.2 *μ*M isoorientin, 3.6 *μ*M orientin, and 4.8 *μ*M rutin. As shown in Table 1, orientin and isoorientin, which are luteolin *C*-glycosylated derivatives, are major flavonoids present in CBS. The inhibitory effects of orientin and isoorientin at a range of 5–30 *μ*M on iNOS and COX-2 expression in LPS-stimulated RAW 264.7 cells were previously reported to be extremely weak [26]. It has been also reported that luteolin *C*-glycosylflavones at 50 *μ*M, including orientin and isoorientin, exhibited no inhibitory effect on LPS-induced NO production in RAW 264.7 cells and had much lower inflammatory effects than those observed with luteolin aglycone and its *O*-glycosides [27]. In addition, vitexin and isovitexin, both of which are apigenin *C*-glycosylflavones, exhibited a very weak inhibitory effect on NO production in LPS-induced RAW 264.7 cells [28]. These results may be explained by the fact that *C*-glycosylation of luteolin and apigenin leads to reduction of anti-inflammatory potential. Quercetin-3-*O*-robinobioside present in the CBS extract is structurally very similar to rutin, although the biological effects of this flavonoid have not reported.

However, the TBS extract was found to be a potent inhibitor of the inflammatory response at 250 *μ*g/mL which contained approximately 17.2 *μ*M of rutin. Lee et al. [29] have reported that rutin at 10 and 30 *μ*M attenuates the production of COX-2, iNOS, and NO in LPS-stimulated RAW 264.7 cells. Furthermore, 20 *μ*M of rutin was a potent reducer of LPS-induced prostaglandin E2 production and COX-2 expression in RAW 264.7 cells [30]. Previous studies have reported that NF-*κ*B and MAPK signaling pathways induce the production of inflammatory mediators and that the NF-*κ*B signaling pathway is closely linked to both I*κ*B-*α* degradation and the phosphorylation of MAPK family members [18, 31]. Yeh et al. [32] have reported that rutin inhibits NF-*κ*B production via suppression of I*κ*B-*α* and MAPK phosphorylation in a mouse model of LPS-induced acute lung injury. Moreover, rutin is known to attenuate cyclophosphamide-induced hepatotoxicity via targeting of the NF-*κ*B and MAPK pathways [33]. In this study, we also found that the TBS extract significantly reduced LPS-induced phosphorylation of I*κ*B-*α* and MAPKs. There is a possibility that the blockade of NF-*κ*B and MAPK signaling pathways may be at least partially caused by rutin in TBS extract. Blocking two inflammatory signaling pathways by rutin in TBS extract could cause potential inhibition of LPS-induced COX-2, iNOS, NO, and cytokine production.

## 5. Conclusions

Anti-inflammatory effects of common and tartary buckwheat sprout extracts rich in flavonoids was comparatively investigated in LPS-stimulated RAW 264.7 and primary peritoneal macrophages. Our findings indicate that TBS extract exhibits more potent inhibition of the production of proinflammatory mediators including cytokines, NO, and iNOS in LPS-stimulated RAW 264.7 macrophages than CBS extract. These outcomes are mediated by the suppression of NF-*κ*B activation by preventing I*κ*B-*α* degradation and MAPK phosphorylation in LPS-stimulated RAW 264.7 macrophages. The TBS extract contained high concentration of rutin as a major flavonoid. Moreover, TBS extract markedly inhibited LPS-induced cytokine release in peritoneal macrophages. Taken together, our results suggest that TBS is a potent anti-inflammatory agent and has potential for development into a therapeutic agent for inflammation-associated disorders.

## Supplementary Material

Supplementary Figure 1.Effect of common and tartary buckwheat sprout extracts on the cell viability of RAW 264.7 cells (A) and peritoneal macrophages (B). Cells were plated in 96 well plate at density of 2 × 10^4^ cells/well for RAW 264.7 cells and 6 × 10^4^ cells/well for peritoneal macrophages. The cells were pretreated with various concentrations of samples for 24 h and cytotoxicity was determined by MTT assay.

## Figures and Tables

**Table 1:** Concentrations (mg/g extracts) of major flavonoids in common and tartary buckwheat sprouts.

Sprout	Orientin	Isoorientin	Vitexin	Isovitexin	Q-3-R	Rutin
Common buckwheat sprout	6.4 ± 0.5^a^	12.9 ± 0.6	12.1 ± 0.1	16.4 ± 0.1	5.5 ± 0.2	11.8 ± 0.5
Tartary buckwheat sprout	ND	ND	ND	ND	ND	42.1 ± 1.0

Concentration of quercetin-3-*O*-robinobioside is expressed as rutin equivalent. ^a^All data are presented as mean ± standard deviation (*n* = 3). Q-3-R: quercetin-3-*O*-robinobioside; ND: not detected.

## Abbreviations

CBS: Common buckwheat sprouts
COX: Cyclooxygenase
DMEM: Dulbecco's modified Eagle's medium
ELISA: Enzyme-linked immunosorbent assay
ERK: Extracellular signal-regulated kinase
FBS: Fetal bovine serum
GAPDH: Glyceraldehyde 3-phosphate dehydrogenase
HPLC: High-performance liquid chromatography
IL: Interleukin
iNOS: Inducible nitric oxide synthase
I*κ*B-*α:*: Inhibitor kappa B-alpha
JNK: c-Jun N-terminal kinase
IFN: Interferon
LPS: Lipopolysaccharide
MAPKs: Mitogen-activated protein kinases
MAPKK: MAPK kinase
MKK4: MAPK kinase 4
MTT: 3-(4,5-dimethylthiazol-2-yl)-2,5-diphenyltetrazolium bromide
NF-*κ*B: Nuclear factor-kappa B
NO: Nitric oxide
SDS: Sodium dodecyl sulfate
TBS: Tartary buckwheat sprouts
TNF: Tumor necrosis factor.
