# Supplementary material for: Comparison of Anti-Inflammatory Effects of Flavonoid-Rich Common and Tartary Buckwheat Sprout Extracts in Lipopolysaccharide-Stimulated RAW 264.7 and Peritoneal Macrophages
Source: Oxid Med Cell Longev. 2017 Aug 27;2017:9658030. doi: 10.1155/2017/9658030 (PMC5591968; doi:10.1155/2017/9658030)
Supplement: Supplementary file 1 — Supplementary Figure 1.Effect of common and tartary buckwheat sprout extracts on the cell viability of RAW 264.7 cells (A) and peritoneal macrophages (B). Cells were plated in 96 well plate at density of 2 × 10^4^ cells/well for RAW 264.7 cells and 6 × 10^4^ cells/well for peritoneal macrophages. The cells were pretreated with various concentrations of samples for 24 h and cytotoxicity was determined by MTT assay. [file 9658030.f1.pdf]

Supplementary Material

Comparison of Anti-inflammatory Effects of Flavonoid-rich Common and Tartary Buckwheat Sprout Extracts in LPS-stimulated RAW 264.7 and Peritoneal Macrophages

Supplementary Figure 1

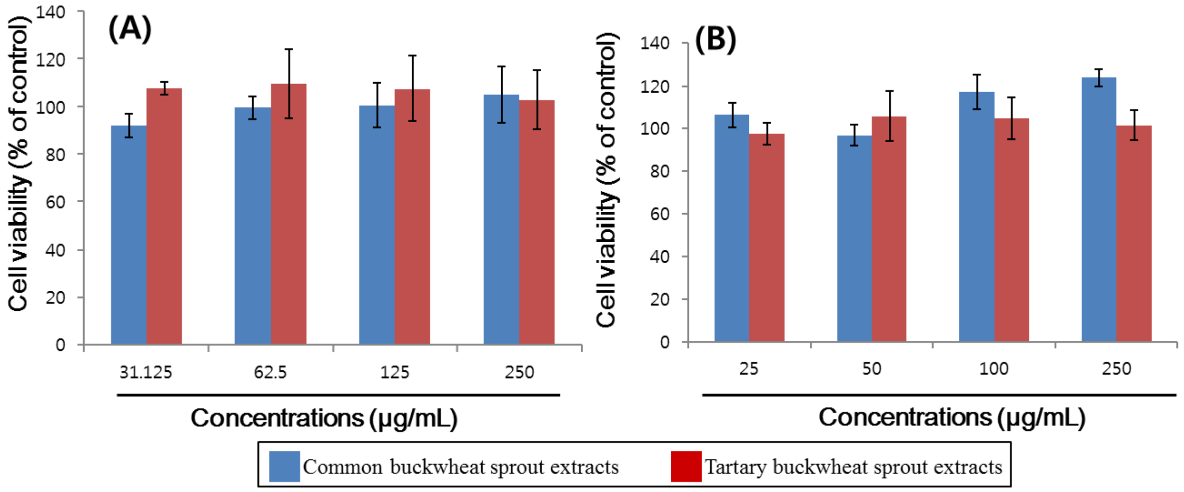

Supp. Figure 1. Effect of common and tartary buckwheat sprout extracts on the cell viability of RAW 264.7 cells **(A)** and peritoneal macrophages **(B)**. Cells were plated in 96 well plate at density of 2×10^4 cells/well for RAW 264.7 cells and 6×10^4 cells/well for peritoneal macrophages. The cells were pretreated with various concentrations of samples for 24 h and cytotoxicity was determined by MTT assay.
